# Cell-derived Newcastle disease virus variant with two amino acid substitutions near cleavage site of F shows favorable traits as oncolytic virus

**DOI:** 10.1016/j.omton.2024.200915

**Published:** 2024-12-06

**Authors:** Marco Huberts, J. Fréderique de Graaf, Daphne Groeneveld, Stefan van Nieuwkoop, Ron A.M. Fouchier, Bernadette G. van den Hoogen

**Affiliations:** 1 Department of Viroscience, Erasmus Medical Centrum, Doctor Molewaterplein 40, 3015 CN Rotterdam, the Netherlands; 2 Department of Immunology, Leids Universitair Medisch Centrum, Albinusdreef 2, 2333 ZA Leiden, the Netherlands

**Keywords:** MT: Regular Issue, Newcastle disease virus, NDV, viro-immunotherapy, pancreatic cancer, embryonated chicken eggs, mammalian cells, human complement system, amino acid substitutions, cleavage site

## Abstract

Newcastle disease virus (NDV) has shown encouraging effectiveness in *in vitro*, *in vivo*, and in early clinical trials as a viro-immunotherapy for pancreatic cancer. Previously, NDV used in clinical trials was produced in embryonated chicken eggs; however, egg-produced viruses are known to be partly neutralized by the human complement system when administered intravenously. Here, an NDV variant (NDV F0) was generated for production in mammalian cells, without passage in eggs. This was achieved by introducing the V-_106_-M and L-_117_-S amino acid substitutions upstream of the cleavage site in the F protein, resulting in rNDV F0-M, rNDV F0-S, and NDV F0-M/S. These viruses can be considered non-virulent as determined with *in vivo* pathogenicity testing and were neutralized less by the human complement system, which is explained by CD46 expression on the viral membrane. The inoculation of 10 pancreatic cancer cell lines demonstrated similar or enhanced replication and cell-killing efficacy of rNDV F0-M/S compared to rNDV F0 and rNDV F0-M. In conclusion, NDV F0 variants with M and S substitutions are non-virulent, effective oncolytic viruses that can be produced in mammalian cells, potentially resulting in a more effective treatment option for pancreatic cancer patients compared to rNDV F0.

## Graphical abstract

**Figure:**
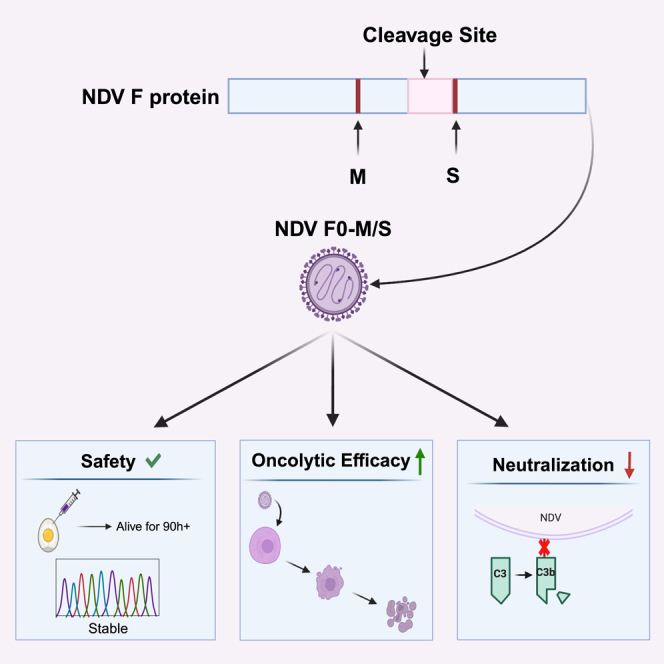

## Teaser

van den Hoogen and colleagues show that introduction of V-_106_-M and L-_117_-S amino acid substitutions in NDV resulted in the ability to produce virus in mammalian cells without the use of embryonated chicken eggs. These substitutions enhanced viral replication and induced cell-killing in pancreatic cancer cell lines while remaining non-virulent.

## Introduction

Viro-immunotherapy is a cancer treatment modality based on oncolytic viruses (OVs) that exert tumor-selective replication and killing and induce potent antitumor immune responses.^1^^,^^2^ OVs take advantage of aberrant antiviral responses in tumor cells, while normal cells remain largely unaffected due to intact early antiviral responses.^3^ An extensively researched OV is Newcastle disease virus (NDV). NDV is a non-segmented, negative sense RNA virus of the family *Paramyxoviridae*, genus Orthoavulavirus, with an avian host under normal circumstances.^4^ The oncolytic activity of NDV was first observed in 1959, when inoculation of Yoshida sarcoma-bearing mice with a virulent Miyadera strain resulted in increased survival.^5^ Since then, a significant number of studies have provided evidence of NDV being a safe and potent oncolytic treatment for a range of tumor types, including pancreatic cancer.^6^^,^^7^^,^^8^^,^^9^ NDV has shown to exert strong direct oncolytic effects in a panel of pancreatic cancer cell lines as well as in *in vivo* models, making NDV a potential treatment modality for pancreatic cancer.^6^^,^^10^^,^^11^^,^^12^

Classification of NDV in lentogenic (avirulent), mesogenic (medium virulent), and velogenic (highly virulent) variants is based on several parameters, including the amino acid sequence of the cleavage site of the fusion (F) protein, which determines by which proteases its cleavage site can be cleaved.^13^ Lentogenic NDV contains a monobasic cleavage site that can be cleaved only by trypsin-like proteases, while mesogenic and velogenic NDV variants have a multibasic cleavage site that can be cleaved by furin-like proteases.^14^ Both lentogenic and mesogenic viruses have been shown to have potent anticancer properties in pre-clinical and clinical settings.^7^^,^^9^^,^^15^ Although mesogenic viruses, such as NDV F3aa, which has three amino acid substitutions in the F protein that result in a multibasic cleavage site, exert increased antitumor effects compared to lentogenic viruses such as NDV F0, they may pose an environmental risk as they could cause severe disease in poultry.^16^^,^^17^ In 2008, mesogenic and velogenic viruses were categorized as select agents by the US Department of Health and Human Services.^18^ Therefore, mainly lentogenic NDV variants are used in viro-immunotherapy studies due to their relatively low virulence in avian species.

Viro-immunotherapy clinical trials with lentogenic and mesogenic NDV have thus far been conducted with virus stocks produced in the allantoic fluid of embryonated chicken eggs.^8^^,^^9^^,^^15^^,^^19^ However, egg-produced NDV may be partially neutralized by the human complement system upon intravenous application.^20^ The neutralizing activity of the complement system reduces the infectivity of NDV and probably necessitates higher doses or repeated injections of egg-produced virus in clinical studies to obtain a therapeutic effect. Viruses produced in mammalian cells will express regulators of complement activity (RCAs), incorporated in their membrane, which renders the virus less sensitive to neutralization by the human complement system.^21^^,^^22^ RCAs are thought to function in a species-specific manner to protect host cells from unregulated complement activation cascades.^23^ For example, viruses produced in Vero cells will have RCA CD46 incorporated in the viral membrane, while viruses produced in HeLa cells will express two RCAs, CD46 and CD55.^20^ Indeed, it has been shown that mammalian cell-produced NDVs were less efficiently neutralized by the human complement system than by egg-produced NDVs.^20^^,^^21^ The use of NDVs produced in mammalian cells and the decreased neutralization by the human complement system might result in higher oncolytic efficacy of the virus in clinical settings.

Here, we report on the generation of NDV variants bearing mutations identified through serial passaging in mammalian cells for which virus stocks could be produced directly in Vero cells to high titers. The virulence, fusogenicity, complement system-mediated neutralization, and oncolytic effect of these variants were assessed.

## Results

### Serial passaging of rNDV F0 resulted in a V-_106_-M substitution in the F protein

**Figure 1:**
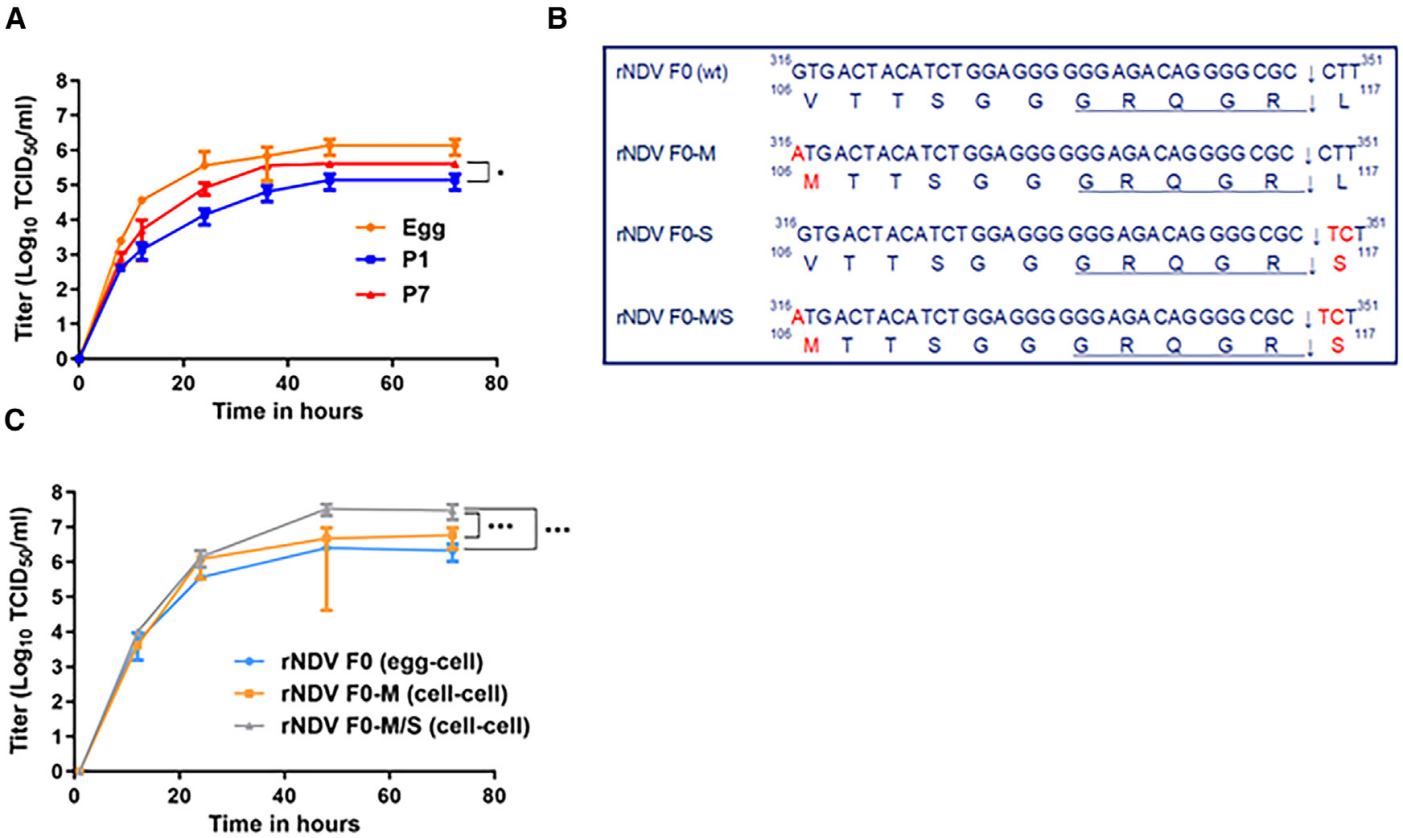
Replication kinetics of serial passaged rNDV F0 in Vero cells and schematic overview of the mutations in the genome of rNDV F0 (A) Replication kinetics of egg-produced and passage (P) 1 and P7 of Vero cell-produced rNDV F0 in Vero cells upon inoculation at an MOI of 0.05. Samples were taken at indicated time points and titrated by endpoint dilution assay in Vero cells. Means and SDs of triplicate titrations are shown. Unpaired t tests were conducted on the AUC of each replication curve. ∗*p* < 0.05. (B) Schematic overview of the observed mutations and introduced mutations in the genome of rNDV F0 to generate rNDV F0-M, rNDV F0-S, and rNDV F0-M/S. (C) Replication kinetics of rNDV F0 (egg-cell), rNDV F0-M (cell-cell), and rNDV F0-M/S (cell-cell) in Vero cells upon inoculation at an MOI of 0.05. Samples were collected at indicated time points and titrated in Vero cells. Means and SDs of triplicate titrations are shown. Statistical analyses were conducted using one-way ANOVA, followed by Tukey’s post hoc multiple comparisons test. ∗∗∗*p* < 0.001.

To generate a mammalian cell-adapted version of recombinant NDV (rNDV) F0, the virus was passaged seven times (P7) in Vero cells after P1 in embryonated chicken eggs. Replication kinetics of the passaged virus demonstrated significantly improved replication of Vero-P7 compared to P1 viruses. Moreover, titers of Vero-P7 rNDV F0 approached similar endpoint titers of rNDV F0 produced in embryonated chicken eggs (Figure 1A). Analysis of the viral genomes of cell-produced rNDV F0 P7 revealed a guanine-to-adenosine substitution (G-_316_-A) 17 bp upstream of the cleavage site of the F protein (Figure 1B), leading to a valine-to-methionine (V-_106_-M) substitution, hereafter called the M substitution. No additional amino acid substitutions were detected in the F protein. However, one amino acid substitution (leucine to proline) at position 4406 (L-4406-P) was identified in the L gene, but this mutation was not investigated further.

### NDV with the M substitution allows production in Vero cells directly after virus rescue in BSR-T7 cells and shows improved replication in Vero cells

An rNDV F0 variant was produced containing this M substitution, resulting in rNDV F0-M. Another rNDV F0 variant was produced containing the L-_117_-S substitution, hereafter called the S substitution, previously described in mesogenic NDV F3aa and NDV-73T, which increased fusogenicity in Vero, HT1080, and HeLa cells and resulted in rNDV F0-S (Figure 1B).^24^^,^^25^ In addition, a virus with both substitutions was produced, resulting in rNDV F0-M/S (Figure 1B). All three variants were generated through site-directed mutagenesis and subsequent molecular cloning.

To test whether eggs could indeed be omitted for the production of virus stocks of NDV, supernatant of BSR-T7 cells transfected with the rescue system for rNDV F0-M, rNDV F0-S, or rNDV F0-M/S was used to inoculate either embryonated chicken eggs or Vero cells. In eggs, rNDV F0-M, rNDV F0-S, and rNDV F0-M/S reached at least a 10-fold higher endpoint titer than rNDV F0 (Table 1). In Vero cells, only rNDV F0-M and rNDV F0-M/S had detectable titers, reaching 1.3 × 10^4^ and 2.2 × 10^6^ median tissue culture infectious dose (TCID_50_)/mL, respectively (Table 1). A subsequent passage in Vero cells of these virus stocks resulted in 1 × 10^7^ and 1 × 10^8^ TCID_50_/mL for rNDV F0-M and rNDV F0-M/S, respectively (Table 1). For rNDV F0 and rNDV F0-S, a preceding passage in eggs was required before virus stocks could be produced in Vero cells. Those viral titers were 3.2 × 10^6^ and 1.0 × 10^7^ TCID_50_/mL for cell-produced rNDV F0 and rNDV F0-S stocks, respectively (Table 1).

**Table 1:** Virus titers obtained with each production method

Virus	Egg	Cell	Cell-cell	Egg-cell
rNDV F0	1 × 10^8^	N.D.	N.P.	3.2 × 10^6^
rNDV F0-M	3.2 × 10^9^	1.3 × 10^4^	1 × 10^7^	N.P.
rNDV F0-S	2.2 × 10^9^	N.D.	N.P.	1.0 × 10^7^
rNDV F0-M/S	5.1 × 10^9^	2.2 × 10^6^	1 × 10^8^	N.P.

Viral titers determined in Vero cells displayed as TCID_50_/mL of virus passaged once in eggs (egg), once in Vero cells (cell), twice in Vero cells (cell-cell), and passaged once in eggs followed by a passage in Vero cells (egg-cell) after virus rescue in BSR-T7 cells. N.D., not detected; N.P., not performed.

Altogether, these data indicated that the M substitution in the F protein allowed direct production of the virus in Vero cells, rendering production of virus stocks independent of passaging in embryonated chicken eggs. Hereafter, virus stocks of rNDV F0-M and rNDV F0-M/S passaged twice in cells are called cell-cell and virus stocks of rNDV F0 passaged once in eggs followed by a passage in cells are labeled as egg-cell.

After generation of the virus stocks, replication kinetics of rNDV F0 (egg-cell), rNDV F0-M (cell-cell), and rNDV F0-M/S (cell-cell) were determined in Vero cells. Analysis of the replication kinetics revealed that rNDV F0-M/S (cell-cell) replicated significantly more efficiently than rNDV F0-M (cell-cell) and rNDV F0 (egg-cell) in Vero cells (Figure 1C), indicating a beneficial role for the S substitution in virus replication.

### NDVs containing the M substitution induce more syncytia formation than the wild-type virus

**Figure 2:**
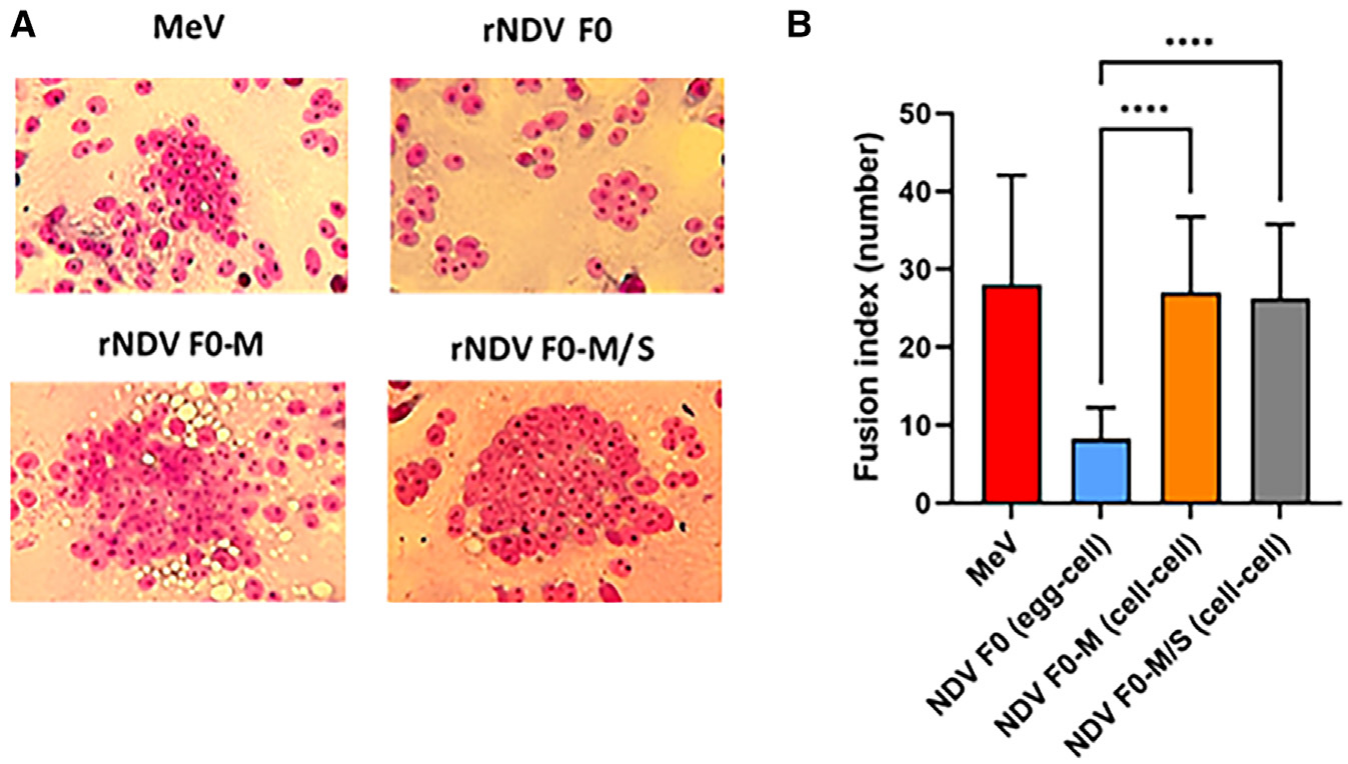
Cell-cell fusion upon inoculation with NDV variants of Vero cells (A) Representative pictures of Giemsa-stained syncytia formed upon inoculation with rNDV F0, rNDV F0-M, rNDV F0-S, and rNDV F0-M/S. (B) The fusion index per virus as determined by counting the number of foci in a syncytium. A total of 30 syncytia were counted, and the average number of foci and SD were plotted. Displayed means and SDs were generated from two independent experiments. Statistical analyses were performed using the Kruskal-Wallis test, followed by Dunn’s post hoc multiple comparisons test. ∗∗∗∗*p* < 0.0001.

The fusogenicity of the NDV variants was assessed 24 h after inoculation of Vero cells with rNDV F0 (egg-cell), rNDV F0-M (cell-cell), rNDV F0-M/S (cell-cell), and the Edmonston strain of measles virus (MeV-Edm), as a positive control. MeV-Edm has been shown to consistently induce syncytia, the formation of large multi-nucleated cell bodies, in various cell lines, including Vero cells.^26^ Vero cells inoculated with cell-produced rNDV F0-M and rNDV F0-M/S had significantly more nuclei per fusion foci compared to cells inoculated with rNDV F0 (egg-cell), while no significant differences in fusogenicity were observed between rNDV F0-M (cell-cell) and rNDV F0-M/S (cell-cell) (Figure 2). This indicates that the M substitution in the F protein of rNDV F0 improved the fusogenicity of the virus.

### M and S substitutions are stable and do not induce changes to the cleavage site of the F protein after serial passaging

To evaluate the stability of the introduced substitutions and to evaluate whether the introduced mutations resulted in additional mutations in the monobasic cleavage site that could lead to a multibasic cleavage site, rNDV F0-M/S (cell-cell) was passaged 10 times in embryonated chicken eggs. Afterward, virus of rNDV F0-M/S (cell-cell) of P1 and P10 were subjected to Illumina sequencing, which resulted in an average genomic coverage of 9,741 reads per nucleotide for P1 and 25,814 reads per nucleotide for P10 of rNDV F0-M/S. Using a variant allele frequency cutoff of 5%, no SNPs were detected at the positions coding for the M and S amino acid substitutions and no additional mutations were observed in or near the cleavage site of the F protein, indicating that the sequence coding for the cleavage site and the M and S amino acids were stable (Table 2). However, after P10, one non-synonymous amino acid substitution, I-_1226_-T, was observed in the polymerase (L) protein of rNDV F0-M/S. The impact of this substitution on viral fitness is unclear.

**Table 2:** Analysis of SNPs in genomes of rNDV F0-M/S upon serial passaging in embryonated chicken eggs

Mutations in P1 and P10	Coverage	VAF, %	AAS	Gene
P1				
A_-6408-_G	11,482	14.37	N.A.	HN (gene start)
G_-8280-_T	6,817	7.07	N.A.	HN/L (intergenic region)
T_-8503-_A	8,344	8.37	synonymous	L protein
P10				
G_-8280-_T	13,507	6.83	N.A.	HN/L (intergenic region)
T_-8503-_A	31,910	51.71	synonymous	L protein
T_-12057-_C	15,010	10.57	I-1226-T	L protein

Full genomes of passage 1 (P1) and P10 of rNDV F0-M/S in embryonated chicken eggs were obtained with Illumina sequencing. The variant allele frequency (VAF) cutoff was set at 5%. AAS, amino acid substitution; N.A., not applicable.

### Recombinant NDV containing M and S substitutions showed improved replication and cell killing in several HPACs compared to the wild-type virus

**Figure 3:**
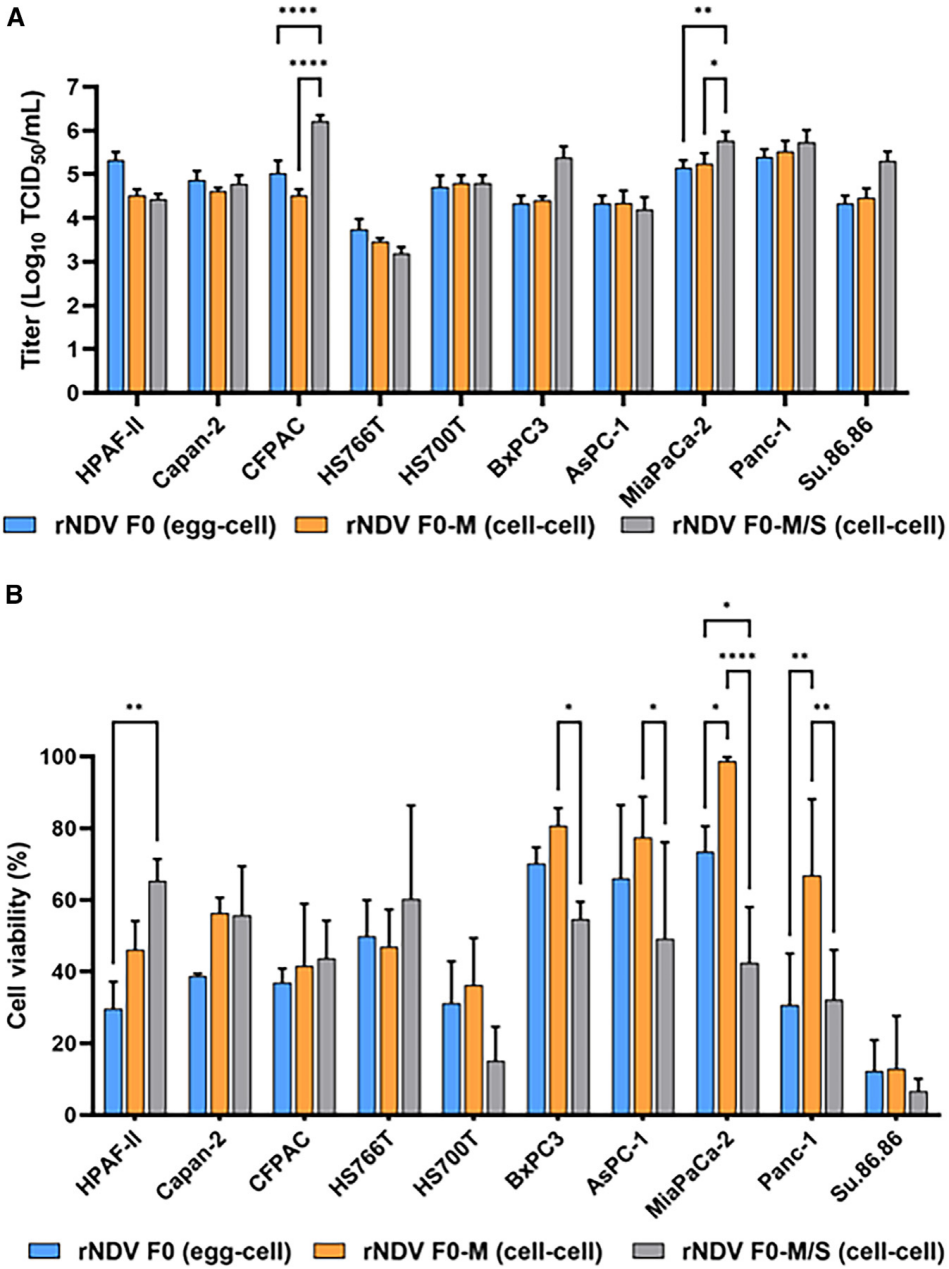
Direct oncolytic effects of NDV F0 (egg-cell), NDV F0-M (cell-cell) and NDV F0-M/S (cell-cell) in HPACs (A) Virus titers in a panel of human pancreatic cancer cell lines 48 h after inoculation at an MOI of 0.1 with rNDV F0 (egg-cell), rNDV F0-M (cell-cell), and rNDV F0-M/S (cell-cell). Experiments were conducted in triplicate, and means and SDs are depicted. Statistical analyses were performed using two-way ANOVA. ∗*p* < 0.05; ∗∗*p* < 0.01; ∗∗∗∗*p* < 0.0001. (B) Cells were inoculated at an MOI of 1 of each virus variant, and cell viability was measured 5 days post-inoculation by using an LDH assay. Data are presented as the percentage of surviving cells compared to mock-treated cells (control) that were considered 100% viable. Experiments were conducted in triplicate, and means and SDs are depicted. Statistical analyses were performed using two-way ANOVA. ∗*p* < 0.05; ∗∗*p* < 0.01; ∗∗∗∗*p* < 0.0001.

Upon the inoculation of 10 different human pancreatic adenocarcinoma cell lines (HPACs) with the cell-grown variants, rNDV F0-M/S (cell-cell) replicated to similar endpoint titers as compared to rNDV F0-M (cell-cell) and rNDV F0 (egg-cell) in several HPACs (Figure 3A). rNDV F0-M/S (cell-cell) replicated to significantly higher endpoint titers than rNDV F0 (egg-cell) only in CFPAC and MIA PaCa-2 (Figure 3A). No significant differences were found between the endpoint titers of rNDV F0-M/S (cell-cell) and rNDV F0 (egg-cell) in eight cell lines (Figure 3B). However, in BxPC-3 and Su.86.86 cells, rNDV F0-M/S (cell-cell) replicated to higher endpoint titers than rNDV F0 (egg-cell), although the difference was not statistically significant. rNDV F0 (egg-cell) replicated to higher endpoint titers than rNDV F0-M/S (cell-cell) or rNDV F0-M (cell-cell) in HPAF-II, and to a lesser extend in HS766T; however, these differences were not significant.

Subsequent assessment of the cell-killing capacity of these NDV variants in the same 10 HPACs cells revealed variability in induced cell killing between the three viruses. No significant differences were found between rNDV F0-M/S (cell-cell) and rNDV F0 (egg-cell) in eight cell lines (Figure 3B). A significant increase in cell killing induced by rNDV F0-M/S compared to rNDV F0 was observed only in MIA PaCa-2 cells. In addition, rNDV F0-M/S (cell-cell)-induced cell killing was significantly higher than that of rNDV F0-M (cell-cell) in Panc-1, MIA PaCa-2, AsPC-1, and BxPC-3 cells. In HPAF-II, rNDV F0 (egg-cell) exhibited a significant increase in cell killing compared to rNDV F0-M/S (cell-cell). These data indicate that, although introduction of the S amino acid substitution did not increase the fusogenicity of NDV F0-M, the presence of both substitutions did increase replication and cell-killing capacity in several HPACs.

### NDV containing the M and S substitutions is non-virulent

To evaluate the effect of the M and S substitutions on the virulence of NDV, a mean death time (MDT) assay was performed. In this assay, the time until the lowest dose of virus becomes lethal for chicken embryos is determined. The virus can be classified as non-virulent if the MDT is longer than 90 h.^16^^,^^27^^,^^28^ In addition to rNDV F0, only rNDV F0-M/S was included in this assay as it exerted more cell killing compared to rNDV F0-M in HPACs, implying increased virulence. The minimum lethal dose was reached at 1 × 10^2^ TCID_50_/mL for rNDV F0-M/S (cell-cell) and rNDV F0 (egg-cell). At this dosage, the induced MDTs of rNDV F0 and rNDV F0-M/S were 104 and 93.3 h, respectively. As the virulence threshold value is set at an MDT of <90 h,^16^ both rNDV F0 and rNDV F0-M/S can be considered non-virulent.

### Cell-produced NDV escapes neutralization by human sera

To evaluate the effect of the of the M and S substitutions on virus neutralization by the mammalian complement system, egg-produced viruses rNDV F0 (egg) and rNDV F0-M/S (egg) and cell-produced viruses rNDV F0 (egg-cell) and rNDV F0-M/S (cell-cell) were incubated with three human sera collected from different anonymous donors. These sera were left untreated or were heat inactivated (HI) to inactivate the complement system. NDV antibody-spiked human sera as a positive control (both HI treated and untreated) neutralized both egg- and cell-produced viruses efficiently, although some variation in neutralization between HI-inactivated and untreated sera was observed (Table 3). None of the viruses were neutralized by treated (HI) human sera. In contrast, all untreated (non-HI) human sera neutralized egg-produced viruses but not cell-produced viruses (Table 3). These data indicate that independent of the presence of the M and S substitutions, NDV variants passaged at least once in cells were neutralized less efficiently by the human complement system compared to egg-produced viruses.

**Table 3:** Virus neutralization titers for egg-grown and cell-grown viruses

Virus	Serum 1	Serum 2	Serum 3	Spiked serum
rNDV F0 (egg)				
non-HI	43 ± 35	53 ± 23	47 ± 31	533.3 ± 184.8
HI	0	0	0	320.0 ± 0
rNDV F0 (egg-cell)				
non-HI	0	0	0	320.0 ± 0
HI	0	0	0	320.0 ± 0
rNDV F0-M/S (egg)				
non-HI	107 ± 46	93 ± 61	47 ± 31	853.3 ± 369.5
HI	0	0	0	80.0 ± 0
rNDV F0-M/S (cell-cell)				
non-HI	0	0	0	853.3 ± 369.5
HI	0	0	0	133.3 ± 46.2

rNDV F0 and rNDV F0-M/S were incubated with heat-inactivated (HI) or non-heat-inactivated (non-HI) human serum. In addition, viruses were incubated with human sera spiked with polyclonal anti-NDV antibody as a positive control. Values indicate the neutralizing titer determined as the highest dilution of serum until NDV infection is detected. Means and SDs of triplicate neutralizing titers are displayed.

### CD46 is strictly present on cell-produced NDV variants

**Figure 4:**
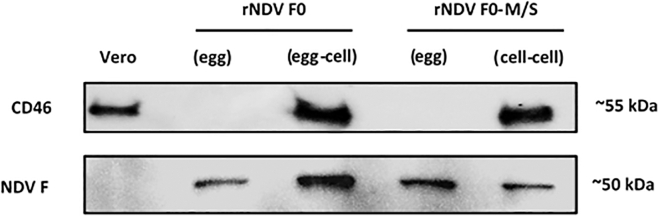
Expression of the CD46 protein by cell-produced NDV Expression of the RCA protein CD46 (∼55 kDa) and the NDV F protein (∼50 kDa) by Vero cells and egg- and cell-produced rNDV F0 and rNDV F0-M/S as detected by western blot using rabbit monoclonal anti-CD46 and chicken polyclonal anti-NDV.

Expression of RCA CD46 on virus particles propagated in Vero cells, which express CD46, is known to be associated with limited virus neutralization by the complement system. Expression of this protein on NDV particles was assessed by means of western blotting. This showed expression of NDV antigens in all samples, but CD46 was only expressed on viruses produced in Vero cells and not on viruses produced in embryonated chicken eggs (Figure 4). These data confirmed that cell-produced rNDV variants, regardless of the presence of M and S substitutions, expressed CD46 on their membranes, which explains the lower neutralization of these cell-produced viruses by the human complement system.

## Discussion

Oncolytic NDV has been shown to be a promising antitumor treatment modality for various cancers. Most clinical trials have used egg-produced NDV, which is known to be partially neutralized by the mammalian complement system.^19^^,^^20^^,^^21^ It has been shown that NDV grown in mammalian cells, including Vero cells, is less prone to complement-mediated neutralization due to the expression of RCA CD46.^20^ Therefore, ideally, virus batches should be produced in mammalian cells, for which Vero cells are the most logical choice as good manufacturing practice Vero cells are approved for vaccine production.^29^

Unfortunately, production of NDV F0 in Vero cells often results in virus titers that are too low for human application. Here, we showed that a virus variant, containing the M substitution in the viral genome near the cleavage site of F, was able to replicate directly in Vero cells after virus rescue.

Previously, we reported on rNDV F3aa-S for which virus stocks could be produced in Vero cells without a preceding egg passage.^28^ However, in the present study, virus stocks for rNDV F0-S could not be produced in Vero cells. This difference between rNDV F0-S and rNDV F3aa-S implies that the ability of NDV variants carrying the S substitution to replicate directly in Vero cells, after virus rescue in BSRT7 cells, is dependent on the composition of the cleavage site. Virus stocks of the NDV F0 variant with both substitutions, M and S, could be produced directly in Vero cells after virus rescue, and this variant replicated slightly better than rNDV F0 or rNDV F0-M in both Vero cells and HPACs. This indicated that the contribution of both amino acid substitutions was necessary for improved replication, independent of the cleavage site motif.

Our data confirmed that viruses produced in Vero cells, with or without the M/S substitutions, express RCA CD46 on their membranes and that these viruses were less efficiently neutralized by the human complement system. Whether this decreased neutralization of the virus contributes to increased oncolytic efficacy can only be assessed in *in vivo* pre-clinical and clinical studies.

The use of NDV in clinical applications could raise concerns about the safety for poultry. The parental NDV F0 virus is the LaSota strain, which is used as a vaccine strain in the poultry industry. Introduction of the M and S substitutions did not change the virulence of the new variant, as confirmed with pathogenicity assays. Additional 10-fold passages of rNDV F0-M/S in embryonated chicken eggs revealed no new amino acid substitutions near the cleavage site of the F protein, showing that the M and S amino acid substitutions did not induce the formation of a multibasic cleavage site. As rNDV F0-M/S had a virulence similar to that of rNDV F0, and because several clinical studies have demonstrated the safety of lentogenic and mesogenic NDV variants in clinical trials, rNDV-M/S should also be safe to use in clinical trials.^8^^,^^9^^,^^15^^,^^19^

The fusion activity of viruses is frequently associated with viral spread and killing of bystander cells, and therefore overall oncolytic effects.^30^ The cell-produced rNDV F0-M and rNDV F0-M/S had increased fusion activity compared to rNDV F0 (egg-cell). However, the fusion activity of rNDV F0-M and rNDV F0-M/S was similar, indicating that the S substitution did not contribute to the increased fusion activity of rNDV F0. This is in contrast to previous studies with rNDV F3aa, where it was shown that rNDV F3aa-S had improved fusion activity compared to rNDV F3aa.^25^ This suggests that the effect of the S substitution on fusion activity is dependent on the presence of a multibasic cleavage site. The influence of the M substitution in rNDV F3aa has not been evaluated, but at least in rNDV F0 the M substitution increased fusogenicity. However, this increased fusogenicity of rNDV F0-M did not correlate with increased replication and induction of cell killing of HPACs, because in several HPACs, rNDV F0-M/S had improved replication efficiency and cell-killing capacity compared to rNDV F0 and rNDV F0-M. Inoculation with rNDV F0-M/S resulted in higher endpoint titers and increased cell killing in MIA PaCa-2 compared to inoculation with rNDV F0 and rNDV F0-M. However, despite significantly increased replication of rNDV F0-M/S, compared to rNDV F0 and rNDV F0-M, in CFPAC cells did not lead to improved cell killing in this cell line. The exact reason for this discrepancy remains unclear.

Overall, rNDV F0-M/S exhibited replication and cell killing comparable to that of rNDV F0 in 8 of 10 pancreatic cancer cell lines, highlighting the consistency of their oncolytic effects and reinforcing the potential of rNDV F0-M/S as an effective therapeutic option.

In conclusion, introduction of the M and S substitutions in the genome of rNDV F0 resulted in a stable, non-virulent OV for which virus stocks can be directly produced in Vero cells after virus rescue in BSR-T7 cells. In addition, rNDV F0-M/S replicated to higher titers in Vero cells and tumor cells than rNDV F0, which not only allows production of higher titer batches but also results in improved virus-induced cell killing. This study showed the potential of this novel NDV variant to be used as a viro-immunotherapy for pancreatic cancer.

## Materials and methods

### Ethics statement

Human serum samples were obtained from three healthy adult volunteers. Written informed consent was obtained from all participants before sample collection. Samples were anonymized to protect participant confidentiality, and data handling complied with applicable data protection laws.

### Cell lines

Vero cells were obtained from the American Type Culture Collection and cultured in DMEM supplemented with 100 U/mL penicillin, 100 U/mL streptomycin, and 2 mM l-glutamine (PSG) and 10% HyClone Characterized Fetal Bovine Serum (FBS). BSRT-7 cells, a derivative of baby hamster kidney (BHK-21) cells stably expressing the T7 RNA polymerase (kind gift of K. Conzelmann),^31^ were cultured in DMEM supplemented with PSG, 10% FBS, 1% non-essential amino acids, and 1% sodium pyruvate. The HPACs were cultured as described previously.^7^ HPAF-II (American Type Culture Collection [ATCC] CRL-1997), Su.86.86 (ATCC CRL-1837), BxPC-3 (ATCC CRL-1687), Panc-1 (ATCC CRL-1469), MIA PaCa-2 (ATCC CRL-1420), and HS766T (ATCC HTB-134) were cultured until P30, while AsPC-1 (ATCC CRL-1682), HS700T (ATCC HTB-147), Capan-2 (ATCC HTB-80), and CFPAC (ATCC CRL-1919) were cultured until P40. All cells were kept at 37°C and 5% CO_2_ in a humidified atmosphere. All media and supplements were purchased from Gibco (Life Technologies).

### Production of lentogenic rNDV F0 in embryonated chicken eggs

The reverse genetics system for the recombinant lentogenic NDV LaSota strain (rNDV F0) has been described before and was kindly provided by Prof. B. Peeters from the Central Veterinary Institute of Wageningen, the Netherlands.^32^ In brief, BSR-T7 cells were transfected with 5 μg full-length pNDV-F0, 2.5 μg pCIneo-NP, 1.25 μg pCIneo-P, and 1.25 μg pCIneo-L using the calcium phosphate precipitation method. After 48 h, 100 μL supernatant was injected into the allantoic fluid of 11-day old embryonated chicken eggs. The embryonated chicken eggs were incubated in a humidified atmosphere in an egg incubator at 37°C for 3 days. Allantoic fluid was harvested, and the infectious titer of the allantoic fluid was determined by endpoint titration in Vero cells, visualized by immunofluorescence assay as described before, calculated using the method of Reed and Muench and expressed as TCID_50_/mL.^10^^,^^33^

### Serial passaging of NDV F0 in Vero cells

One million Vero cells were seeded per well in a 6-well plate. The next day, cells were inoculated with egg-produced rNDV F0 at an MOI of 0.1 in DMEM supplemented with PSG and 1 μg/mL l-(tosylamido-2-phenyl) ethyl chloromethyl ketone (TPCK)-treated trypsin (T1426, Sigma-Aldrich), and the medium was refreshed every 2 days. When 60%–70% cytopathic effects (CPEs) were observed, the cells were scraped, and the cell-supernatant mixture was harvested and frozen at −80°C for one freeze/thaw cycle. The infectious titers were determined in Vero cells as described above. For the next passage, the harvested virus was used to inoculate Vero cells at an MOI of 0.1. In total, P7 were performed, and 200 μL supernatant of virus P1 and P7 was collected and used for RNA isolation and MinION sequencing of full-length rNDV genomes.

### Sequencing of full-length genomes with MinION

RNA extraction was performed using the High Pure RNA isolation kit (Roche Diagnostics) according to the manufacturer’s instructions and eluted in 50 μL elution buffer. Copy DNA (cDNA) was produced using the Superscript IV Reverse Transcriptase Kit (Invitrogen, Thermo Fisher) according to the manufacturer’s instructions. The cDNA was amplified by PCR using PFU Ultra II Fusion HS NA polymerase (Agilent Technologies), and seven overlapping amplicons of the NDV genome were generated using their corresponding primer sets as published previously.^24^ Following the PCR assay, the products were purified using AMPure XP beads. Sample concentrations were measured with the Qubit dsDNA HS Assay Kit (Thermo Fisher) on a Qubit fluorometer (Thermo Fisher); 700 ng DNA was used per sample for library preparation. Samples of P1 and P7 of rNDV F0 in Vero cells were barcoded using the 1D Native Barcoding Genomic DNA Kit (EXP-NBD104 and SQK-LSK109) according to the manufacturer’s instructions and sequenced using an R9.4 FLO-MIN106 flow cell (Oxford Nanopore Technologies) on a GridION Mk1 (Oxford Nanopore Technologies) for 16 h.

### Sequencing of full-length genomes with Illumina

Preparation of samples was similar to that of MinION sequencing. Library preparation of samples of P1 and P10 of rNDV F0-M/S in embryonated chicken eggs was performed using the KAPA HyperPlus library preparation kit (Roche) according to the manufacturer’s instructions and sequenced using the Illumina Nextseq 2000 (2 × 300 cycles) platform.

### Site-directed mutagenesis and molecular cloning

Site-directed mutagenesis was performed as described before.^7^^,^^32^ Briefly, mutations were introduced in a subclone containing the P, M, and F genes of rNDV F0 in the pGEM 5Zf backbone. rNDV F0-M was generated by introducing a G-_316_-A nucleotide substitution upstream of the cleavage site of the F gene of rNDV F0, resulting in the V-_106_-M amino acid substitution. rNDV F0-S was generated by introducing C-_349_-T and T-_350_-C mutations in the F gene, resulting in the L-_117_-S amino acid substitution. rNDV F0-M/S was generated by introducing G-_316_-A and both C-_349_-T and T-_350_-C mutations, resulting in the V-_106_-M and L-_117_-S amino acid substitution in the F protein, respectively. The subclone containing the site-directed mutations was placed back into the full-length cDNA clone of rNDV F0 (see Table 1). The full-length constructs were sequenced using MinION to exclude incidental mutations that could arise during the cloning process.

### Production of recombinant NDV stocks in Vero cells

rNDV variants were rescued by transfecting BSR-T7 cells as described above. After 2 days, BSR-T7 cells were scraped and co-cultured with Vero cells in DMEM with PSG and 1 μg/mL TPCK-treated trypsin, and medium was refreshed every 2 days. Upon the appearance of CPE, the cells were harvested together with the supernatant and frozen at −80°C for one freeze/thaw cycle. To create a P1 stock in Vero cells, the cell-supernatant mixture from the BSR-T7-Vero co-culture was centrifuged for 10 min at 1,000 rpm and 1 mL supernatant was added to 5 mL Vero cells in DMEM with PSG and 1 μg/mL TPCK-treated trypsin. Cells were incubated until CPE was observed, and the cell-supernatant mixture was stored at 80°C for one freeze/thaw cycle. The infectious titers were determined in Vero cells as described above.

### Replication kinetics

One million Vero cells were seeded per well in a 6-well plate. The next day, cells were inoculated with rNDV variants at an MOI of 0.05. After 1 h, the cells were washed three times with PBS, and DMEM with PSG and 1 μg/mL TPCK-treated trypsin was added. At each time point, 100 μL supernatant was collected and stored with 100 μL 50% sucrose (w/w) at −80°C. Infectious titers of collected samples were determined in Vero cells as described above. For replication in HPACs, 24-well plates were seeded with 2 × 10^5^ cells/well and inoculated with virus at an MOI of 0.1. After 1 h, cells were washed three times with PBS, and subsequently, RPMI 1640 media supplemented with PSG and 1 μg/mL TPCK-treated trypsin was added. Forty-eight hours after washing, the supernatant was collected, and infectious titers were determined in Vero cells as described above.

### Virus neutralization assay

Virus neutralization assays were performed as described previously.^34^ Briefly, sera received from three healthy anonymous human donors were left untreated (non-HI) or were HI for 30 min at 56°C. Additionally, one of the human sera was spiked with polyclonal anti-NDV as a positive control. The initial 10-fold dilution of sera and the subsequent 2-fold serial dilutions were mixed 1:1 with 100 TCID_50_/mL of either egg- or cell-produced variants of rNDV F0 and rNDV F0-M/S. After incubation for 1 h at 37°C, mixtures were transferred to 96-well plates containing 20,000 Vero cells/well. After another 1-h incubation at 37°C, the plates were washed with PBS and 200 μL fresh DMEM media supplemented with 1 μg/mL TPCK-treated trypsin per well was added. Virus neutralization titers were assessed 5 days after inoculation by determining the dilution of serum until virus infection was detected.

### Fusion assay

Vero cells (7.5 × 10^5^ cells/well) were seeded in 6-well plates. The next day, cells were inoculated at an MOI of 1 of various rNDVs or MeV-Edm as a positive control in DMEM with PSG. Seven hours post-inoculation, 1 μg/mL TPCK-treated trypsin was added to the culture to assist in the cleavage of the F protein. Cells were fixated and stained with Giemsa 24 h post-inoculation. The fusion index was determined by averaging the number of nuclei of 30 fusion foci.

### MDT assay of embryonated chicken eggs

Ten-fold dilution series of the viruses was diluted in PBS. For each dilution, groups of six 10-day-old specific pathogen-free embryonated chicken eggs were inoculated with 100 μL virus into the allantoic fluid. The eggs were incubated at 37°C in a humidified incubator and were checked every 8 h for 7 days. The time that each embryo was first observed dead was determined by shining light on the air sac of the embryonated egg and characterized by collapsed blood vessels and an immobilized embryo. The highest dilution that killed all the embryos was considered the minimum lethal dose. The MDT was calculated as the mean time in hours for the minimum virus dose to kill all embryos.

### Cell viability assay

HPACs were plated in triplicate at 2 × 10^4^ cells/well in a 96-well plate and either mock inoculated with RPMI or inoculated with rNDV at an MOI of 1. Five days post-inoculation, cells were washed once with PBS and lysed with 100 μL 0.9% Triton X-100 for 45 min at 37°C. Viability was determined by assessing lactate dehydrogenase (LDH) release from lysed cells using the CytoTox 96 Non-Radioactive Cytotoxicity Assay (Promega) following the manufacturer’s instructions. Cell viability is presented as the percentage of absorption at 450 nm of inoculated cells versus mock inoculated cells, which were considered to have a viability of 100%.

### Western blot assay

A confluent T75 flask of Vero cells and 200 μL egg- and cell-produced rNDV F0 and rNDV F0-MS were lysed with radioimmunoprecipitation assay (RIPA) buffer (Pierce Biotechnology). RIPA was supplemented with complete Mini EDTA-free protease inhibitors (Roche). Protein concentrations were assessed using a bicinchoninic acid assay protein assay kit (Pierce Biotechnology). Virus-isolated protein samples, normalized based on viral titer, and 1 μg Vero-isolated protein samples were loaded and run on an SDS-PAGE with 10% polyacrylamide (Bio-Rad) for 2 h at 50 V. Afterward, proteins were transferred onto polyvinylidene difluoride membranes (Millipore) for 1 h at 200 mA. Membranes were blocked overnight using PBS + 0.1% Tween (PBST) and 10% milk. Subsequently, membranes were incubated overnight with rabbit monoclonal anti-CD46 (Abcam, catalog no. ab231984, 1:250) or chicken polyclonal anti-NDV (Abcam, catalog no. ab34402, 1:2,000). Membranes were washed with PBST and incubated with secondary antibodies: horseradish peroxidase (HRP)-conjugated polyclonal swine anti-rabbit antibody (Agilent Technologies, catalog no. PO217, 1:2,000) and HRP-conjugated polyclonal anti-chicken (Abcam, catalog no. ab97135, 1:2,000) for 1 h. Membranes were extensively washed for 2 h using PBST, developed with chemiluminescence (Amersham ECL), and imaged using the ChemiDoc Touch Imaging System (Bio-Rad).

### Statistical analysis

All statistical analyses were performed using GraphPad Prism 10. Each dataset was first assessed for normality using the Shapiro-Wilk test to determine the appropriate statistical analysis. Replication kinetics were quantified by calculating the area under the curve (AUC). Differences between AUCs were analyzed through an unpaired t test or a one-way ANOVA, depending on the number of groups to evaluate. For datasets that did not follow a normal distribution, a Kruskal-Wallis test was performed to evaluate overall group differences, followed by a Dunn’s multiple comparisons test for post hoc analysis. For normally distributed data consisting of two independent variables across three (or more) groups, a two-way ANOVA was performed. Upon obtaining statistically significant ANOVA results, Tukey’s multiple comparisons test was performed as a post hoc analysis. Statistical significance was set at *p* < 0.05 for all analyses.

## Data and code availability

The data and biological materials that support the findings of this study are available from the corresponding author upon request.

## Acknowledgments

We would like to acknowledge the support from the Dutch Foundation OAK (Overleven met Alvleesklier kanker) from the Netherlands, grants #16.01 and #19.10, and Netherlands NWO-TTW grant #15414 (NWO-domein Toegepaste en Technische Wetenschappen). The graphical abstract and figures were created with BioRender (https://BioRender.com/d60m404).

## Author contributions

M.H., J.F.d.G., D.G., S.v.N., and B.G.v.d.H. designed the experiments and analyzed the data. M.H., J.F.d.G., D.G., and S.v.N performed the experiments. R.A.M.F. and B.G.v.d.H. provided experiment resources and technical support. All authors contributed to the writing and reviewing of the manuscript.

## Declaration of interests

The authors declare no competing interests.
